# Post orgasmic illness syndrome: what do we know till now?

**DOI:** 10.1186/s12610-019-0093-7

**Published:** 2019-09-03

**Authors:** Maher Abdessater, Sandra Elias, Elie Mikhael, Abdalla Alhammadi, Sebastien Beley

**Affiliations:** 1 Groupe Hospitalier Diaconesses - Croix Saint Simon, 125 rue d’Avron, 75020 Paris, France; 2 Institut Gustave ROUSSY, Villejuif, France; 3 Groupement hospitalier Eaubonne, Montmorency (Hôpital Simone Veil), Eaubonne, France

**Keywords:** Post orgasmic illness syndrome (POIS), Pathophysiology, Symptoms and complaints, Differential diagnoses, Work up, Management, Syndrome de maladie post-orgasmique (SMPO), Physiopathologie, Symptômes, Diagnostics différentiels, Investigations, Traitements

## Abstract

**Background:**

Peri orgasmic dysfunctions are very rare and little information exists on their diagnosis and treatment. One of these conditions is post-orgasmic illness syndrome (POIS), manifesting by a debilitating cluster of symptoms affecting men within seconds, minutes, or hours after ejaculation. The aim of this article is to do a thorough literature review about POIS, in order to elucidate the pathophysiology, the diagnosis and the management of this rare disease.

**Results:**

Updated literature review on Pubmed was done, using the following terms: “orgasm illness”, “post-orgasmic” and “postorgasmic illness syndrome”. The references of the 17 identified publications were also reviewed for additional 8 relevant articles that were all included in the results.

POIS has 5 preliminary diagnostic criteria and criterion 1 has 7 described clusters. Pathophysiological hypotheses include: immunological phenomenon (most relevant), opioid-like withdrawal, neuroendocrine response, transient deregulation of the autonomic nervous system, hypersensitivity and disordered cytokines. Differential diagnoses include: chronic prostatitis, orgasmolepsy, benign orgasmic cephalgia, sneezing and rhinorrhea, postcoital dysphoria, post-coital asthma and rhinitis. Patients have been symptomatically treated with antihistamines, non-steroidal anti-inflammatory drugs, selective serotonin reuptake inhibitors, and benzodiazepines. A trial of hyposensitization therapy with autologous semen was successful.

**Conclusion:**

POIS is a rare condition that is underdiagnosed, most probably because of its unclear pathophysiology leading to a lack of treatment options. Further studies are warranted to investigate the prevalence, pathophysiology, and management of this debilitating condition.

## Résumé

**Contexte:**

Les dysfonctions péri-orgasmiques sont très rares et nous disposons de peu d’informations sur leurs diagnostic et traitement. L’une de ces affections est le syndrome de la maladie post-orgasmique (SMPO), qui se manifeste par un groupe de symptômes handicapants affectant les hommes durant les secondes, minutes ou heures après l’éjaculation. Le but de cet article est de faire une revue approfondie de la littérature sur le SMPO, afin d’élucider sa physiopathologie et son diagnostic pour mieux gérer cette maladie rare.

**Résultats:**

Nous avons réalisé une revue de la littérature actualisée sur Pubmed en utilisant les termes suivants: “orgasm illness”, “post-orgasmic” and “postorgasmic illness syndrome”. Les références des 17 publications identifiées ont également été examinées pour inclure 8 autres articles pertinents dans les résultats.

Le SMPO est défini par 5 critères diagnostiques préliminaires et le critère 1 comprend 7 groupes de symptômes. Les hypothèses physiopathologiques comprennent: un phénomène immunologique (le plus pertinent), un sevrage des opioïdes endogènes, une réponse neuroendocrine, une dérégulation transitoire du système nerveux autonome, une hypersensibilité et des désordres des cytokines. Les diagnostics différentiels incluent: la prostatite chronique, l’orgasmolepsie, la céphalée orgasmique bénigne, l’éternuement et la rhinorrhée, la dysphorie post-coïtale, l’asthme post-coïtal et la rhinite. Les traitements n’ont été que symptomatiques par antihistaminiques, anti-inflammatoires non stéroïdiens, inhibiteurs sélectifs de la recapture de la sérotonine et benzodiazépines. Un essai thérapeutique de désensibilisation avec du sperme autologue a été couronné de succès.

**Conclusion:**

Le SMPO est une maladie rare sous-diagnostiquée, probablement à cause de sa physiopathologie peu claire, conduisant à un manque d’options thérapeutiques. Des études supplémentaires sont nécessaires pour étudier la prévalence, la physiopathologie et la prise en charge de cette maladie débilitante.

## Introduction

Peri orgasmic dysfunctions are very rare; this is why little information exists on their diagnosis and treatment. One of these conditions is post-orgasmic illness syndrome (POIS), with around sixty cases described in the literature over the last three decades.

This rare but debilitating syndrome consisting of a cluster of post ejaculatory symptoms, mainly in men, was first described in 2002 by Waldinger and Schweitzer who reported two cases [1]. Men with POIS became ill few seconds, minutes or hours after ejaculation whether during intercourse, masturbation or even spontaneous nocturnal ejaculation. The complaints started with flu-like symptoms followed by cognitive disorders that lasted for about 5 to 7 days, and reappeared after the next ejaculation [1]. After the second publication of Waldinger on 45 cases [2], few articles described this disease through a small number of case reports. The aim of our work is to do a thorough literature review about POIS, in order to elucidate the diagnosis and management of this rare disease.

## Material and methods

We did an updated literature review on Pubmed using the following terms: “orgasm illness”, “post-orgasmic” and “postorgasmic illness syndrome”. Studies of any design and from any language were accepted whatever the date of the publication was. We identified seventeen pertinent publications and included them all in our review. In addition, the references of these identified publications were reviewed for additional eight articles that were also included in our results.

## Results

### Symptoms and complaints

Using data from 57 reported cases of men with POIS, from which Waldinger et al. published 47 [1, 2], we found that the presentation of POIS was highly variable: concentration difficulties, extreme fatigue, exhaustion and fever, body warmth, perspiration, shivering, mood disturbances, irritability and memory difficulties [1–8]. This is why we think that the discoverers of this syndrome, after further research, suggested 5 preliminary diagnostic criteria to assess the large spectrum of this disease [2]. These criteria are presented in Table 1. The authors further stratified the symptoms in Criterion 1 into 7 clusters, as described by the patients’ own words (Table 2).

**Table 1:** Preliminary diagnostic criteria of POIS [2]

CRITERIA	CLINICAL PRESENTATION
Criterion 1	One or more of the following symptoms: Sensation of a flu-like state, extreme fatigue or exhaustion, weakness of musculature, experiences of feverishness or perspiration, mood disturbances and/or irritability, memory difficulties, concentration problems, incoherent speech, congestion of nose or watery nose, itching eyes.
Criterion 2	All symptoms occur immediately, soon (e.g., seconds, minutes) or few hours after ejaculation that is initiated by coitus, and/or masturbation, and/or spontaneously (e.g., during sleep).
Criterion 3	Symptoms occur always or nearly always, e.g., in more than 90% of ejaculation events.
Criterion 4	Most of these symptoms last for about 2 to 7 days.
Criterion 5	The symptoms disappear spontaneously.

**Table 2:** Categorization of POIS Criterion 1 clusters [2]

CLUSTERS	SYMPTOMS	CATEGORIES
Cluster 1	Extreme fatigue, exhausted, palpitations, problems finding words, incoherent speech, dysarthria, concentration difficulties, quickly irritated, cannot stand noise, photophobia, depressed mood.	General
Cluster 2	Feverish, extreme warmth, perspiration, shivery, ill with flu, feeling sick, feeling cold.	Flu-like
Cluster 3	Headache, foggy feeling in the head, heavy feeling in the head	Head
Cluster 4	Burning, red injected eyes, blurred vision, watery, irritating, itching eyes, painful eyes.	Eyes
Cluster 5	Congested nose, watery, runny nose, sneezing.	Nose
Cluster 6	Dirty taste in mouth, dry mouth, sore throat, tickling cough, hoarse voice	Throat
Cluster 7	Muscle tension in back or neck, muscle weakness, pain muscles, heavy legs, stiff muscles	Muscle

They also noted that manifestation of POIS varies in the intensity, durations, sort of symptoms and their order of appearance, and identified a primary type of POIS that appears from the first ejaculation in puberty or adolescence, and a secondary type in which POIS manifests later in life [2].

For years, and since the first definition of POIS, all the authors used Waldinger‘s criteria to diagnose and report their cases. However, in 2019, Strashny published a self-report study, to assess for the first time, the validity of these criteria [9]. His study included 127 men with self-reported POIS but only 20% of these men reported having been diagnosed by a healthcare provider. Almost all the respondents who have completed the survey fulfilled a majority of Waldinger’s criteria. However, 44% of these patients did not fulfill Criterion 3, because they did not always experience symptoms in one of the ejaculatory settings (sex, masturbation, or nocturnal emission). Consequently, Strashny proposed a possible improvement of Criterion 3 which is to amend it to “In at least one ejaculatory setting” instead of “all of them” [9]. He also proposed to reconsider a refinement of Criterion 1 because symptoms are so varied and there does not appear to be a single symptom cluster that defines POIS [9]. Notwithstanding, respondents were not examined by a clinician to verify their answers, which is a major limitation of Strashny’s study.

### Pathophysiology

The pathophysiology of POIS is not clear yet and it has been explored by few studies so far; hypotheses include: an immunological phenomenon [2–5], an opioid-like withdrawal [6], a neuroendocrine response [7], a transient deregulation of the autonomic nervous system [8], a hypersensitivity and disordered cytokines [10, 11].

Waldinger et al. who published the widest case series in the literature, supported their hypothesis as an immune modulated mechanism by the 88% positive skin-prick tests on patients with POIS, using very diluted samples of their own semen [3]. Therefore, they concluded that POIS is the result of a patient’s type I and type IV allergy to his own semen and to his mucosal epithelium of the urinary tract and the seminal fluid. They attributed this conclusion to the fact that POIS symptoms did not occur during sexual activities without ejaculation [2, 3].

Furthermore, they postulated that these immunologic reactions are due multiple close contacts during ejaculations between seminal peptides and T lymphocytes. The origin of these peptides is either the disrupted urethral mucosal cells, or the autologous semen. They are then transported by dendritic cells to the paracortex of lymph nodes where the naïve T cells activation and expansion is produced after interaction with seminal fluid antigens, leading to the hypersensitivity reaction. This secondary hypothesis was supported by the lack of local genital skin reaction after ejaculation, the occurrence of multiple systemic complaints, the incidence of the disease before and after sterilization (so it is not related to spermatozoa) and the successes of hyposensitization treatment [2, 3].

Defending the same hypothesis, Kim et al. confirmed the presence of serum semen-specific IgE in their patient with POIS [4].

Jiang et al. also reported an atopic constitution in their patient. However, they found that there was no evidence of semen-specific IgE antibodies in men with POIS and positive skin reactions to autologous semen. Instead, they suggested that it may be caused by opioid withdrawal, due to similarity in manifestations. This was attributed to the consumption of large quantities of endogenous opioids by the mechanism of orgasm, resulting in symptoms similar to opioid withdrawal. So they concluded that chemical imbalances in the brain might be the physiological basis for POIS with psychological conditions serving as risk factors [6].

In contrast to these hypotheses, Depreux et al. had negative results on immunoblotting and western blot of their POIS patient’s autologous semen [12].

Based on the symptoms improvement of the patients after administration of prophylactic diclofenac, Ashby and Goldmeier proposed that POIS is triggered by a disordered cytokine or neuroendocrine response [7].

Finally, Bignami et al. considered that POIS is the manifestation of a transient dysregulation of the autonomic nervous system since it is well known that ejaculation triggers a “vegetative storm” with an increase in sympathetic activity and a release of norepinephrine [8].

Of note, many authors described a lifelong premature ejaculation (PE) among their POIS patients [2, 6, 8]. PE in these men is probably induced by forced abstinence and low frequency of sexual activity [2]. These patients also wanted to reduce ejaculation frequency as much as they can despite strong desire to sexual relationship, to avoid the disturbing consequences of ejaculation on their life and work [3]. However, none of the authors discussed whether autonomic imbalance may account for PE since they considered it as an associate symptom to POIS and not a phenotype of this syndrome [2, 6, 8].

### Differential diagnoses

#### Chronic prostatitis or chronic pelvic pain

When the diagnosis is not obvious with negative lab tests, these conditions can manifest as flu-like symptoms, weakness and pain of the muscle localized to the perineum or suprapubic area. However, symptoms last longer than those of POIS, fever can be documented and pain on ejaculation with dysuria is the first reported complaint [13, 14].

#### Post-orgasmic cataplexy (orgasmolepsy)

It is a sudden onset of weakness with orgasm, symptoms can mimic cluster 7 of POIS but they typically last for less than 30 s with complete loss of muscle control. The proposed mechanism for this disorder is neurological and the treatment is anticataplectic drugs, including antidepressants or sodium oxybate [15].

#### Orgasm-associated headaches or benign orgasmic cephalgia

Mimicking cluster 3 of criterion 1 of POIS, they are explosive, bilateral and precipitated by orgasm. Their duration range from several minutes to few hours. Magnetic resonance imaging (MRI) followed by magnetic resonance angiography (MRA) should be done to exclude an intracranial lesion or hemorrhage (arteriovenous malformation). No recommendations exist concerning the pharmacologic treatment for postorgasm headache, but trying an antimigraine medication or a pretreatment with propranolol seem to give good results. However, with patients who do not improve on these medications, we can try indomethacin or a calcium channel antagonist such as verapamil. Methysergide or valproic acid can be given as a last resort [16].

#### Sneezing and rhinorrhea

These symptoms occur immediately after orgasm and are non-responsive to antihistamines. The hypothetical pathophysiology of this mechanism is “the activation of one part of the parasympathetic system by stimuli that usually activate a different branch of the parasympathetic system” [17]. The treatment can be topical application of nasal decongestant oxymetazoline hydrochloride 0.05% or of the anesthetic agent 1% tetracaine and 2% ephedrine sulfate to the nostrils before intercourse [18]. Although these symptoms resemble to cluster 5, their isolated character allows us to rule out POIS.

#### Postcoital dysphoria (PCD) or postcoital psychological symptoms (PPS)

Crying, depression and dysphoria despite satisfactory sexual intercourse have been reported to occur with and without orgasm and therefore cannot be considered as a true post-orgasmic phenomenon. PCD can include tearfulness and feelings of melancholy, depression, anxiety, agitation, or aggression which can mimic the psychological burden of POIS; this is why we mentioned it in our review, although symptoms last only up to an hour after sexual intercourse [19].

#### Post-coital asthma and rhinitis

We do not know so far the real mechanism by which sexual intercourse can precipitate exercise-induced asthma or rhinitis. Sex-related emotional excitement and anxiety, which are associated with autonomic imbalance with parasympathetic over activity, may be responsible of this entity; it is probably caused by mast cell mediators released due the cholinergic stimulation. This diagnosis can be differentiated from POIS by the fact that it can be triggered by any cause of emotional excitement or anxiety even without sexual activity. It can be managed by metered dose of inhaled salbutamol before sexual intercourse [20].

### The social load of POIS

POIS has severe mental and psychosocial consequences; the sexual life of men with POIS is totally disturbed: decreased intercourse frequency despite natural desire, abstaining from intercourse and refraining from masturbation or intercourse to the lowest possible level. This behavior is due to fear of ejaculation and the associated debilitating symptoms [2, 6, 7, 21].

Patients plan their intercourse to avoid the consequences of ejaculation on their daily essential activities (work or study for example), because they suffer from a decrease in concentration, alertness and physical capacity [2, 7]. Young patients are hesitant in seeking for the romantic partner, this reluctance is due to the fear of women refusal due to intercourse abstaining [22].

The partners of patients with POIS are also negatively affected [9, 22]. Most men expressed feelings of guilt concerning their relationship with their sexual partner who were not satisfied; some of these relationships ended with divorce due to patients’ strategy of abstinence or avoidance of sexual intercourse [2].

### Work up

Complete medical history, including existing allergies, sexually transmitted diseases, any history of prostatitis or other diseases mimicking POIS, must be taken with a detailed review of lower urinary tract symptoms.

Neuropsychiatric evaluation seeking for possible neurological or psychiatric disorders should be done. Sexual function interview focusing on ejaculatory disorders and the relationship with the partner should be recorded. The duration of symptoms must be well noted since it is an essential factor of the thorough history in making differential diagnosis [2, 7, 12, 23].

Careful physical exam needs to be done, including a digital rectal examination (DRE) after a midstream urine sample has been collected for urine dipstick, microscopic analysis and culture [2, 7, 12, 23].

Patients should undergo routine tests, including full blood count, serum electrolytes and creatinine, liver function tests and hormonal check (follicle stimulating hormone, luteinizing hormone, prolactin, and testosterone) [2, 4, 6, 7, 12].

Brain and medullar MRI may be needed to rule out an intracranial lesion or hemorrhage (arteriovenous malformation) that might lead to a headache after ejaculation [23].

In addition, Bignami et al. did perineal electromyography (EMG), neurovegetative tests and cystoscopy for their patients in order to rule out any uncommon urological condition [8].

Waldinger et al. [2, 3] performed a protocolized intracutaneous skin-prick test with extremely diluted semen of the patient himself in order to objectify the skin reaction after inoculation of the semen, with the potential risk of provocation of the POIS symptoms and even a generalized anaphylactic shock that necessitates intensive care unit. This is why the semen should be reduced to extremely low concentrations (1:40000). Even though the study had the limitation of unmatched age control group, 88% of the patient had a positive skin prick, which makes this test a potential diagnostic tool of POIS.

### Management

Since POIS is a rare underdiagnosed condition, it has no recognized treatment modalities; POIS patients have been treated with antihistamines, selective serotonin reuptake inhibitors, and benzodiazepines [8, 11, 24].

Nonsteroidal anti-inflammatory medication (diclofenac) was successful (up to 80% improvement) and allowed the patient to increase his sexual frequency from 2 to 4 times a month [7], but this same therapy failed in other patients [10]. Jiang et al. also prescribed diclofenac 75 mg 1–2 h prior to sexual activities with orgasm, to be continued twice daily for 24–48 h [6]. Prednisone and tramadol are other treatment options [11].

Considering the successful outcomes of hyposensitization therapy in clinical allergic diseases, hyposensitization therapy could have therapeutic effects in patients with POIS in whom allergies are a dominant etiologic factor. Waldinger et al. supported this hypothesis and reported the improvement of POIS symptoms by the hyposensitization treatment with subcutaneous autologous semen in two Dutch patients [1]. This protocol was developed by Marcus Meinardi who intensified the hyposensitization by passing from extremely diluted autologous semen to higher concentrations of autologous semen [2, 3]. They reported 60 and 90% improvement of POIS complaints at 31 and 15 months, respectively for their first two patients [1].

Kim et al. performed intralymphatic immunotherapy with autologous semen instead of subcutaneous delivery on a Korean male with POIS: using ultrasound guidance and a 25-gauge needle, autologous semen was aseptically injected into an inguinal lymph node at a dilution of 1:40,000. Then, the concentration was increased by 3-fold, as in a previous study by Waldinger. After 15 months, all POIS- related symptoms except sore throat and urinary symptoms were alleviated and their durations were shortened [4].

However, limitations of hyposensitization treatment include a lack of healthy male controls for the autologous semen skin test results.

Finally, numerous anecdotal therapies have been suggested to be efficacious in improving POIS symptoms, including niacin, olive leaf, fenugreek, saw palmetto, and wobenzyme [25].

## Conclusion

POIS has a significant effect on sufferers and their partners in terms of symptoms and quality of life. This rare but debilitating condition deserves further investigations in order to elucidate its real pathophysiology that will allow us to find the adequate treatment. This is not easy at all, due to lack of an evidence-based definition and the rarity of the disease.

The first step has been accomplished and the condition has been recognized by the National Institutes of Health; nevertheless, the introduction of an official definition of POIS is needed especially after the study of Strashny who proposed amendments to the initial definition of the disease [9]. This could lower the barriers to the recognition of POIS by other medical organizations in order to increase the support for its research.

Furthermore, researches on the efficacy and safety of hyposensitization therapy and other possible treatment alternatives could be done in large multicenter clinical trials, allowing much more progress in the diagnosis and the management of this disease in the upcoming years.

## Data Availability

Not applicable

## Abbreviations

DRE: Digital rectal exam
EMG: Electromyography
MRA: Magnetic resonance angiography
MRI: Magnetic resonance imaging
PCD: Postcoital dysphoria
PE: Premature ejaculation
POIS: Post-orgasmic illness syndrome
PPS: Postcoital psychological symptoms
